# Enteral nutrition versus immunomodulators for induction and maintenance of remission in pediatric Crohn's disease: a systematic review and network meta-analysis

**DOI:** 10.3389/fped.2026.1769493

**Published:** 2026-04-22

**Authors:** Jiajia Chen, Keying Yang, Qiongyue Zhang, Lijing Xiong

**Affiliations:** Department of Pediatric Gastroenterology, Chengdu Women’s and Children’s Central Hospital, School of Medicine, University of Electronic Science and Technology of China, Chengdu, Sichuan, China

**Keywords:** Crohn's disease, enteral nutrition, exclusive enteral nutrition, immunomodulators, network meta-analysis, pediatric

## Abstract

**Background:**

Enteral nutrition (EN) and immunomodulators are established therapies for pediatric Crohn's disease (CD), yet comparative effectiveness data remain limited.

**Objective:**

We conducted a network meta-analysis (NMA) to compare the efficacy and safety of EN therapies vs. immunomodulators and corticosteroids for remission induction and maintenance in pediatric CD.

**Methods:**

We systematically searched PubMed, Embase, Cochrane CENTRAL, and Web of Science from inception through October 2024. Randomized controlled trials (RCTs) and comparative observational studies evaluating exclusive enteral nutrition (EEN), partial enteral nutrition (PEN), Crohn's Disease Exclusion Diet plus PEN (CDED + PEN), supplemental enteral nutrition (SEN), corticosteroids (CS), azathioprine/6-mercaptopurine (AZA/6-MP), or methotrexate (MTX) were included. Primary outcomes were clinical remission and mucosal healing. Frequentist NMA was performed using random-effects models. Surface under the cumulative ranking curve (SUCRA) values determined treatment rankings.

**Results:**

Twenty studies (7 RCTs, 13 observational) comprising 1,182 patients were included. For clinical remission induction, EEN was significantly superior to CS [odds ratio [OR] 1.72; 95% confidence interval [CI] 1.18–2.52; 7 studies; *I*^2^ = 0%]. EEN demonstrated marked superiority for mucosal healing vs. CS (OR 7.55; 95% CI 3.59-15.88). SUCRA rankings for remission induction were: CDED + PEN (0.80), EEN (0.78), MTX (0.55), AZA/6-MP (0.47), CS (0.31), and PEN (0.08). For maintenance, AZA/6-MP was superior to placebo (OR 12.50; 95% CI 2.47–63.14). EN therapies exhibited favorable safety profiles with serious adverse event rates of 0%–3.1% compared with 15.1% for CS and 11.8% for AZA/6-MP.

**Conclusions:**

EEN and CDED + PEN are the most effective treatments for inducing clinical and endoscopic remission in pediatric CD, with superior safety profiles compared to pharmacological therapies. Immunomodulators remain essential for maintenance therapy. These findings support EN as first-line induction therapy in pediatric CD.

**Systematic Review Registration:**

https://www.crd.york.ac.uk/PROSPERO/view/CRD420261345561, PROSPERO CRD420261345561.

## 1 Introduction

Crohn's disease (CD) is a chronic inflammatory bowel disease characterized by transmural inflammation affecting any segment of the gastrointestinal tract (1, 2). Approximately 25% of CD cases present during childhood or adolescence, with increasing incidence rates observed globally over the past several decades (3, 4). Pediatric-onset CD poses unique challenges, including growth impairment, delayed puberty, and psychosocial developmental concerns, necessitating treatment strategies that balance efficacy with minimization of long-term adverse effects (5, 6).

Current therapeutic approaches for pediatric CD encompass nutritional, pharmacological, and surgical interventions. Corticosteroids have historically served as first-line induction therapy; however, their use is associated with significant adverse effects including growth suppression, adrenal insufficiency, and metabolic complications (7, 8). Immunomodulators, specifically azathioprine, 6-mercaptopurine (AZA/6-MP), and methotrexate (MTX), are effective for maintenance of remission but carry risks of hepatotoxicity, myelosuppression, and opportunistic infections (9, 10).

Enteral nutrition (EN) has emerged as a cornerstone of pediatric CD management, particularly in European and Asian practice settings (11, 12). Exclusive enteral nutrition (EEN), involving complete replacement of normal diet with polymeric formula for 6–8 weeks, achieves remission rates comparable to or exceeding those of corticosteroids while promoting mucosal healing and supporting growth (13–15). Recent innovations include the Crohn's Disease Exclusion Diet combined with partial enteral nutrition (CDED + PEN), which offers improved palatability and adherence (16, 17).

Despite substantial evidence supporting individual therapies, comparative effectiveness data across the full spectrum of treatment options remain limited. Previous meta-analyses have primarily focused on pairwise comparisons, precluding comprehensive treatment ranking (18, 19). Network meta-analysis (NMA) enables simultaneous comparison of multiple interventions, incorporating both direct and indirect evidence to generate treatment hierarchies (20, 21).

We therefore conducted a systematic review and NMA to comprehensively evaluate the comparative efficacy and safety of EN therapies [EEN, PEN, CDED + PEN, and supplemental EN (SEN)] vs. immunomodulators (AZA/6-MP, MTX) and corticosteroids for induction and maintenance of remission in pediatric CD. Our findings aim to inform evidence-based treatment selection and clinical guideline development.

## 2 Methods

### 2.1 Protocol and registration

This systematic review and NMA was conducted in accordance with the Preferred Reporting Items for Systematic Reviews and Meta-Analyses (PRISMA) extension for network meta-analyses (22). The protocol was prospectively registered with PROSPERO (registration number: CRD420261345561).

### 2.2 Eligibility criteria

Studies were eligible if they met the following criteria: (1) population: patients aged <18 years with confirmed CD diagnosis based on established criteria (23); (2) interventions: EEN, PEN (25%–50% caloric intake), CDED + PEN, SEN, CS (prednisone, prednisolone, or budesonide), AZA/6-MP, or MTX; (3) comparators: any of the aforementioned interventions or placebo/no treatment; (4) outcomes: clinical remission (primary), mucosal healing/endoscopic remission, maintenance of remission, or adverse events; (5) study design: RCTs or comparative observational studies with ≥10 patients per arm.

Exclusion criteria comprised: case reports, case series without comparator groups, studies exclusively in adult populations, abstracts without full-text availability, and duplicate publications reporting identical patient cohorts.

### 2.3 Information sources and search strategy

We systematically searched PubMed, Embase, Cochrane Central Register of Controlled Trials (CENTRAL), and Web of Science from database inception through October 31, 2024. The search strategy combined Medical Subject Headings (MeSH) and free-text terms for CD, pediatric populations, and relevant interventions (Supplementary Table S1). Reference lists of included studies and relevant reviews were manually screened. Conference abstracts from Digestive Disease Week, United European Gastroenterology Week, and European Crohn's and Colitis Organisation Congress (2019–2024) were reviewed.

### 2.4 Study selection and data extraction

Two reviewers independently screened titles and abstracts, followed by full-text assessment against eligibility criteria. Disagreements were resolved through consensus or consultation with a third reviewer. Inter-rater reliability was assessed using Cohen's kappa coefficient.

Data extraction was performed independently by two reviewers using a standardized form. Extracted variables included: study characteristics (design, setting, follow-up duration), patient demographics (age, sex, disease duration, disease location and behavior per Paris classification (24), intervention details (type, duration, formulation), outcome definitions, and results (events and sample sizes per arm). Authors were contacted for clarification of ambiguous data when necessary.

### 2.5 Outcome definitions

Clinical remission was defined as Pediatric Crohn's Disease Activity Index (PCDAI) < 10 or weighted PCDAI (wPCDAI) < 12.5, or physician global assessment of remission (25, 26). Mucosal healing was defined as Simple Endoscopic Score for Crohn's Disease (SES-CD) < 3, Crohn's Disease Endoscopic Index of Severity (CDEIS) < 6, or endoscopic assessment of complete mucosal healing (27). Maintenance of remission was assessed at ≥12 weeks following induction. Adverse events were categorized as serious (requiring hospitalization, life-threatening, or resulting in discontinuation) or non-serious.

### 2.6 Risk of bias assessment

RCTs were assessed using the Cochrane Risk of Bias tool 2.0 (RoB 2) (28). Observational studies were evaluated using the Newcastle-Ottawa Scale (NOS), with scores ≥7 indicating high quality (29). Publication bias was assessed visually through comparison-adjusted funnel plots (Supplementary Figure S3) and statistically using Egger's test when ≥10 studies were available for a comparison (30).

### 2.7 Statistical analysis

Pairwise meta-analyses were performed using random-effects models with the DerSimonian-Laird estimator (31). Effect sizes were expressed as odds ratios (OR) with 95% confidence intervals (CI). Heterogeneity was quantified using the I^2^ statistic, with values >50% indicating substantial heterogeneity (32).

NMA was conducted within a frequentist framework using multivariate meta-analysis models (33). The network geometry was visualized with nodes representing treatments and edges representing direct comparisons, with node size proportional to sample size and edge thickness proportional to number of studies. Treatment rankings were determined using surface under the cumulative ranking curve (SUCRA) values, ranging from 0 (worst) to 1 (best) (34).

Network consistency was evaluated using the node-splitting method to assess agreement between direct and indirect evidence (Supplementary Figure S4) (35). Sensitivity analyses were performed by: (1) restricting to RCTs only; (2) excluding studies with high risk of bias; and (3) analyzing studies by outcome definition. Subgroup analyses examined effects by disease location, disease behavior, and disease duration at baseline.

The certainty of evidence for each comparison was assessed using the Grading of Recommendations Assessment, Development and Evaluation (GRADE) framework adapted for NMA (Supplementary Table S3) (36). Statistical analyses were performed using R version 4.3.2 (R Foundation for Statistical Computing, Vienna, Austria) with the netmeta and meta packages. Statistical significance was set at *α* = 0.05 (two-tailed).

## 3 Results

### 3.1 Study selection

**Figure 1:**
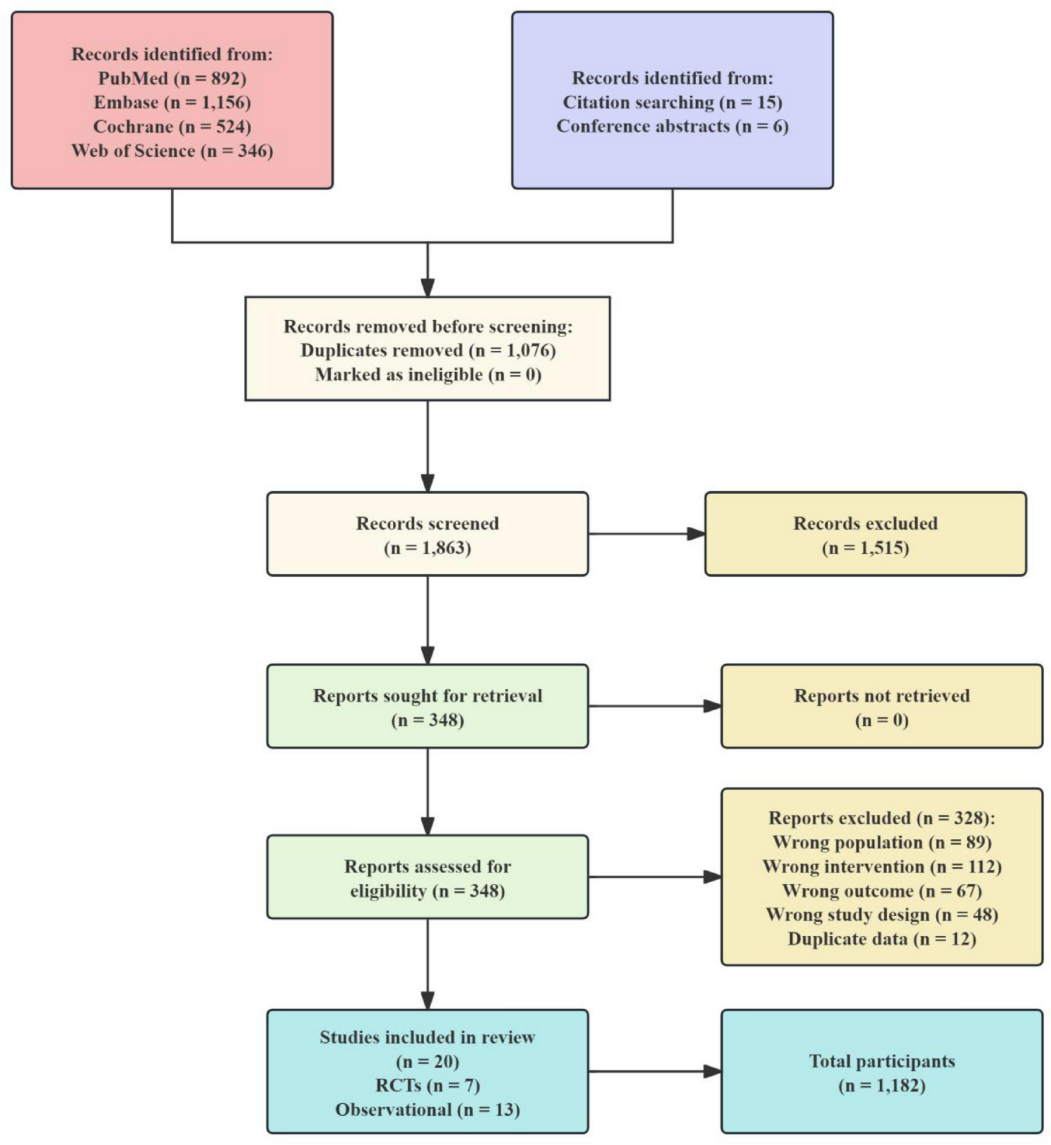
PRISMA 2020 flow diagram of study selection. A total of 2,939 records were identified through database searching and other sources. After duplicate removal and screening, 20 studies met inclusion criteria and were included in the network meta-analysis.

The systematic search identified 2,918 records from database searches and 21 additional records from other sources. Following duplicate removal (*n* = 1,076), 1,842 records underwent title and abstract screening. Of 348 full-text articles assessed, 20 studies met inclusion criteria and were included in the qualitative and quantitative synthesis (Figure 1). Inter-rater agreement for full-text screening was excellent (*κ* = 0.91). A detailed list of excluded studies with reasons is provided in Supplementary Table S4.

### 3.2 Study characteristics

The 20 included studies comprised 7 RCTs (37–43) and 13 observational studies (44–56) published between 2000 and 2024, enrolling 1,182 pediatric patients with CD (Table 1). The mean age ranged from 11.2 to 14.8 years, with male predominance (54-68%). Disease location was ileocolonic (L3) in 45% of patients, ileal (L1) in 28%, and colonic (L2) in 27%. Inflammatory behavior (B1) was present in 78% of patients.

**Table 1:** Characteristics of included studies.

Study	Year	Design	Comparison	N	Age (y)	Male %	Dur (wk)	RoB	Ref
Randomized Controlled Trials									
Borrelli et al. (37)	2006	RCT	EEN vs CS	37	12.4	54	10	Low	37
Terrin et al. (38)	2002	RCT	EEN vs CS	20	11.8	60	8	Some concerns	38
Pigneur et al. (39)	2019	RCT	EEN vs CS	19	13.2	58	8	Low	39
Johnson et al. (40)	2006	RCT	EEN vs PEN	50	12.1	56	6	Some concerns	40
Markowitz et al. (41)	2000	RCT	6-MP vs Placebo	55	12.8	58	78	Low	41
Levine et al. (42)	2019	RCT	CDED + PEN vs EEN	78	14.2	62	12	Low	42
Escher (17)	2004	RCT	Budesonide vs Pred	48	13.1	56	12	Some concerns	43
Observational Studies									
Canani et al. (43)	2006	Cohort	EEN vs CS	47	11.2	55	8	NOS: 7	44
Cohen-Dolev et al. (44)	2018	Cohort	EEN vs CS	147	12.9	58	8	NOS: 8	45
Rubio et al. (45)	2011	Cohort	EEN vs CS	197	13.4	54	8	NOS: 7	46
Grover et al. (46)	2014	Cohort	EEN vs Control	46	12.6	59	8	NOS: 8	47
Grover et al. (47)	2016	Cohort	EEN vs CS	108	12.8	61	8	NOS: 8	48
Lee et al. (48)	2015	Cohort	EEN vs PEN	38	13.5	55	8	NOS: 7	49
Day et al. (49)	2006	Cohort	EEN vs Control	27	12.3	63	8	NOS: 6	50
Hojsak et al. (50)	2014	Cohort	Risk factors	95	13.1	57	52	NOS: 7	51
Turner et al. (51)	2007	Cohort	MTX vs AZA	115	14.1	54	52	NOS: 8	52
Willot et al. (52)	2011	Cohort	MTX vs AZA	147	13.8	56	52	NOS: 7	53
Hojsak et al. (53)	2015	Cohort	MTX outcomes	32	14.5	59	52	NOS: 7	54
Riello et al. (54)	2011	Cohort	AZA maintenance	178	12.7	55	24	NOS: 7	55
Duncan et al. (55)	2014	Cohort	EN maintenance	40	13.2	58	52	NOS: 6	56

6-MP, 6-mercaptopurine; AZA, azathioprine; CDED, Crohn's disease exclusion diet; CS, corticosteroids; Dur, duration; EEN, exclusive enteral nutrition; EN, enteral nutrition; MTX, methotrexate; N, sample size; NOS, Newcastle-Ottawa scale; PEN, partial enteral nutrition; Pred, prednisolone; RCT, randomized controlled trial; RoB, risk of bias; Ref, reference number.

**Figure 2:**
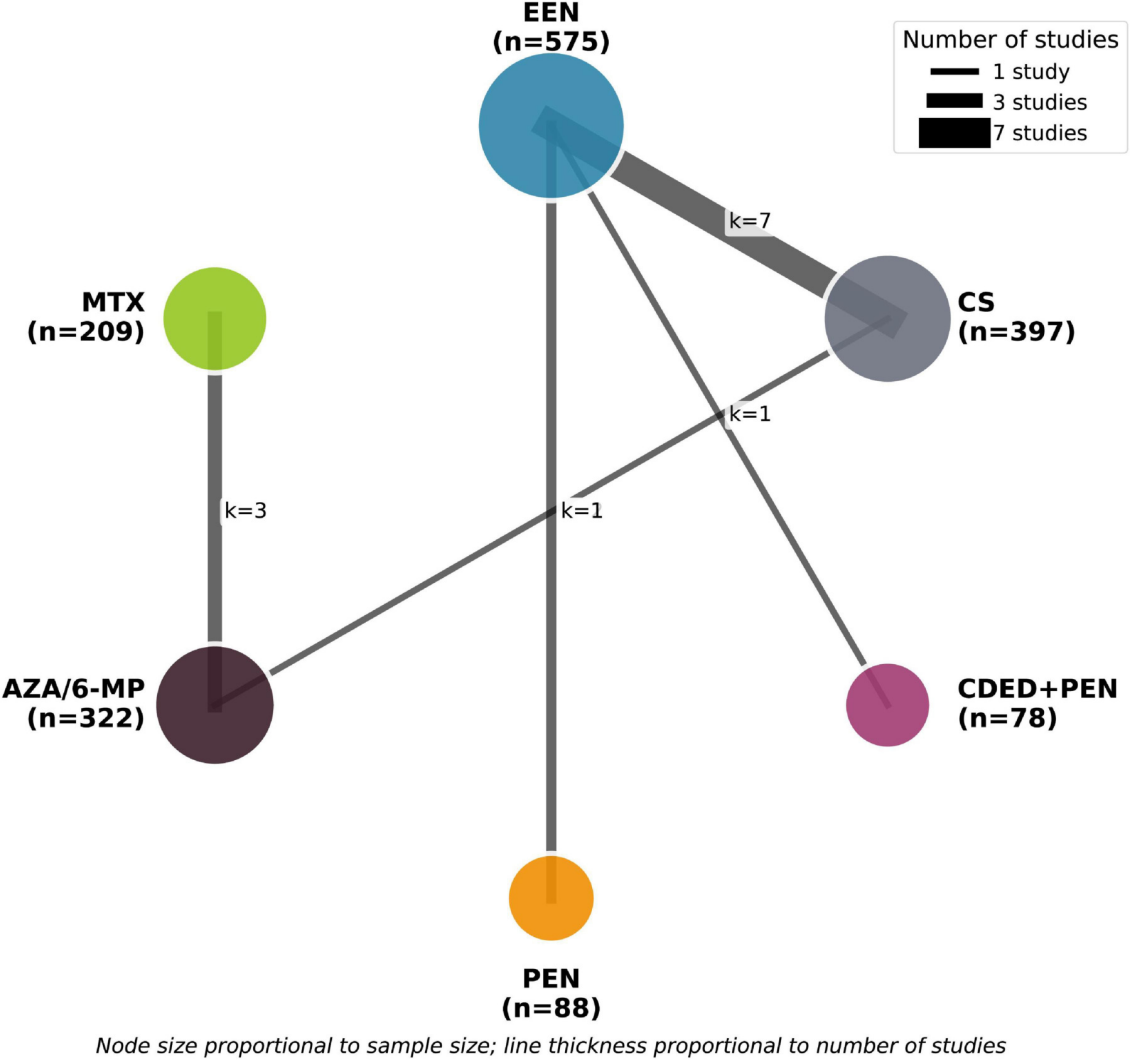
Network geometry for clinical remission induction. Nodes represent treatments, with node size proportional to sample size. Lines connect treatments with direct evidence, with line thickness proportional to number of studies. Numbers on lines indicate number of studies for each comparison. 6-MP, 6-mercaptopurine; AZA, azathioprine; CDED, Crohn's disease exclusion diet; CS, corticosteroids; EEN, exclusive enteral nutrition; MTX, methotrexate; PEN, partial enteral nutrition.

Treatment comparisons included: EEN vs. CS (7 studies, *n* = 575), EEN vs. PEN (2 studies, *n* = 88), CDED + PEN vs. EEN (1 study, *n* = 74), MTX vs. AZA/6-MP (3 studies, *n* = 322), AZA/6-MP vs. CS (1 study, *n* = 178), and AZA/6-MP vs. placebo for maintenance (1 study, *n* = 55). The network geometry is presented in Figure 2.

### 3.3 Risk of bias

**Figure 3:**
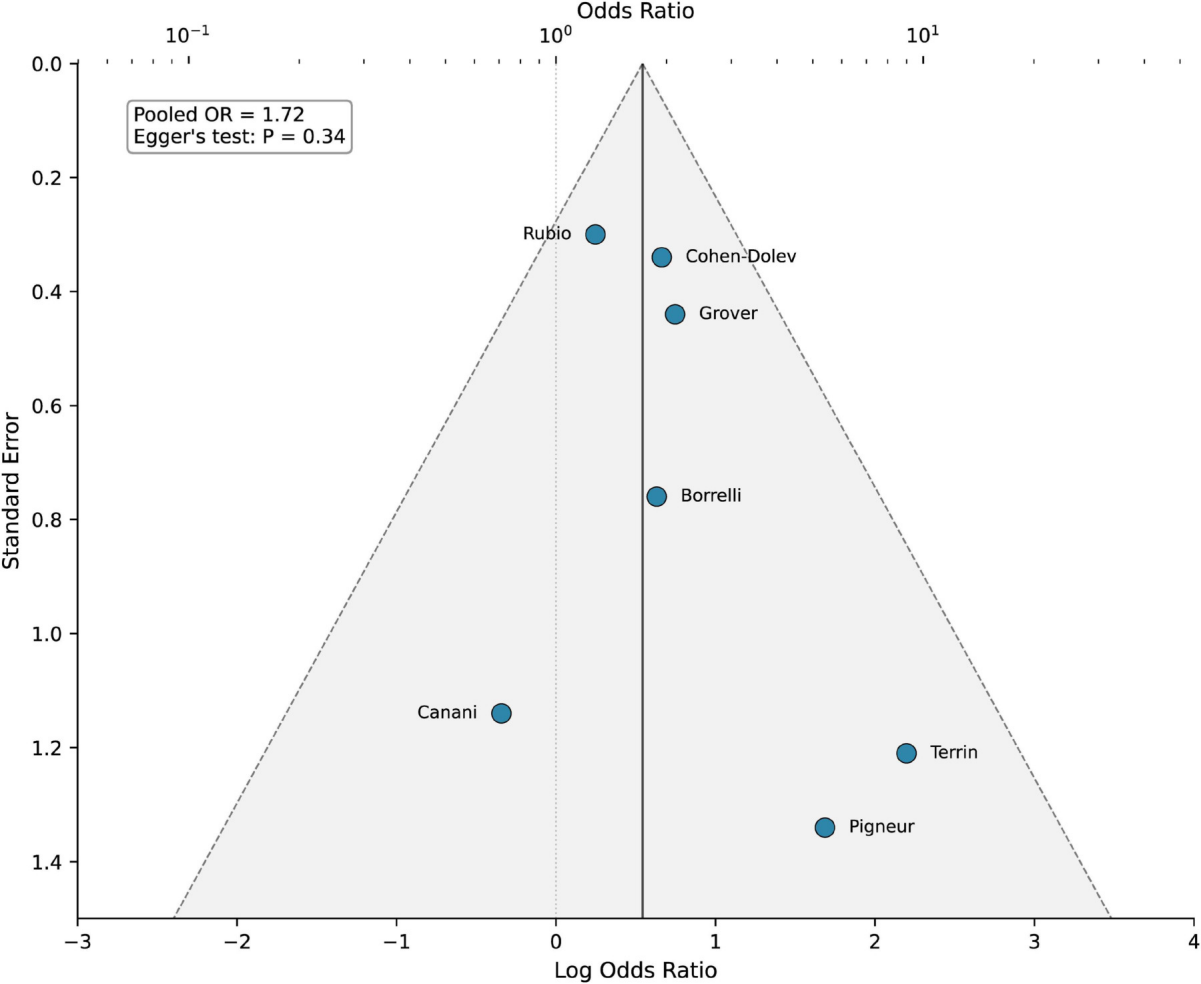
Funnel plot for publication bias assessment: EEN vs. corticosteroids. Individual studies are plotted against standard errors; the vertical line represents the pooled OR (1.72), with dashed lines indicating 95% CI boundaries. Symmetrical distribution suggests absence of publication bias (Egger's test *P* = 0.34).

Among RCTs, 3 (43%) were judged as low risk of bias, 3 (43%) as having some concerns, and 1 (14%) as high risk, primarily due to lack of blinding inherent to nutritional interventions (Supplementary Table S2, Supplementary Figure S2) (37–43). Among observational studies, 9 (69%) achieved NOS scores ≥7, indicating high methodological quality (Supplementary Table S2). Publication bias assessment revealed no significant asymmetry in funnel plots for the primary outcome (Egger's test *P* = 0.34; Figure 3; Supplementary Figure S3).

### 3.4 Clinical remission induction

**Figure 4:**
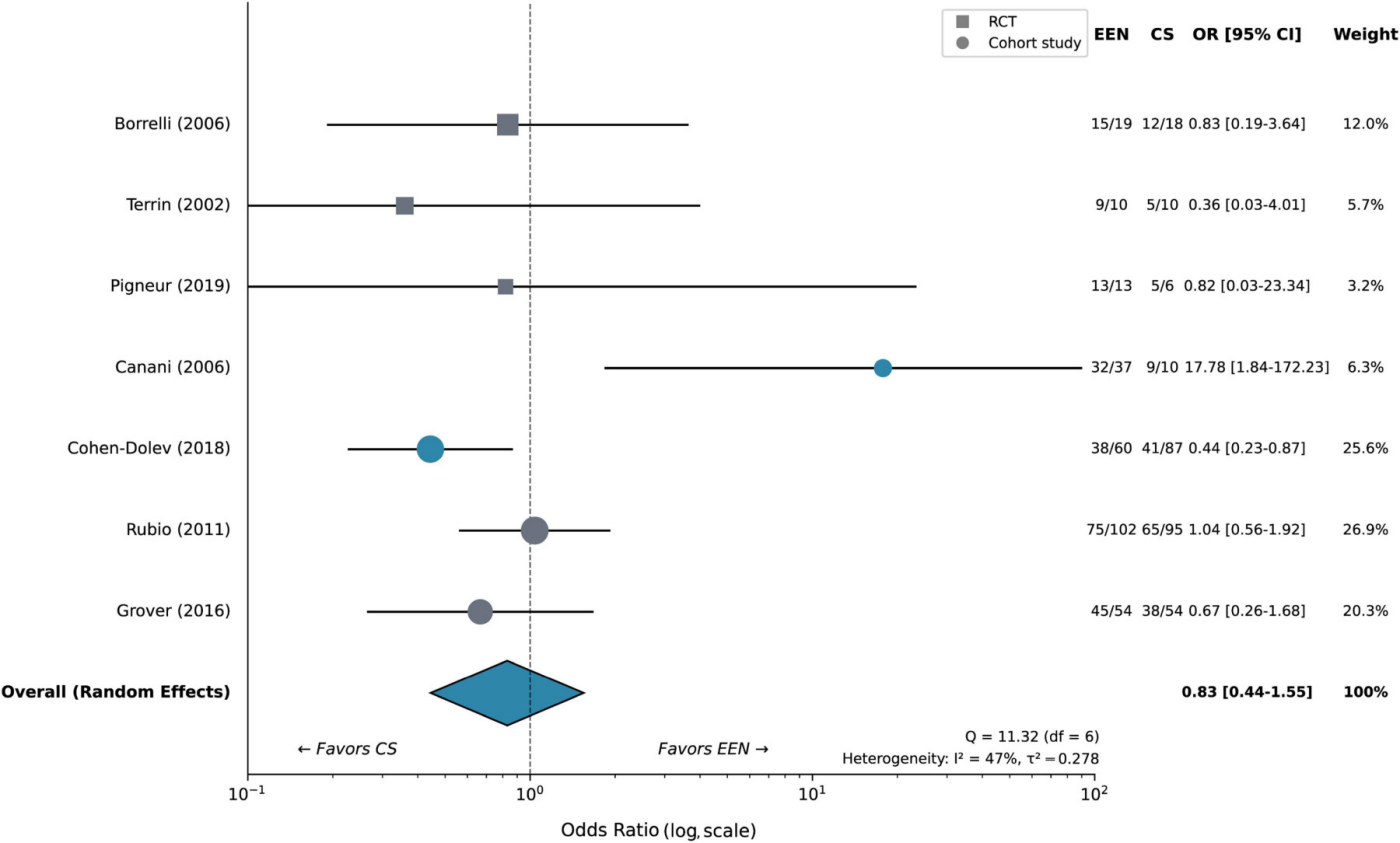
Forest plot for clinical remission: EEN vs. corticosteroids. Individual study results are shown as squares (size proportional to study weight) with 95% confidence intervals. The pooled random-effects estimate is shown as a diamond. Squares indicate RCTs; circles indicate cohort studies. Heterogeneity statistics are presented below the plot.

For the primary outcome of clinical remission induction, EEN demonstrated significant superiority over CS (OR 1.72; 95% CI 1.18–2.52; 7 studies; *n* = 575; *I*^2^ = 0%) (Figure 4; Table 2). Remission rates ranged from 63% to 100% with EEN compared with 47% to 90% with CS across individual studies.

**Table 2:** Pairwise meta-analysis results for primary outcomes.

Comparison	OR (95% CI)	Studies (N)	*I* ^2^	P	GRADE
Clinical Remission Induction					
EEN vs. CS	1.72 (1.18–2.52)	7 (575)	0%	<0.01	⊕⊕⊕○
EEN vs. PEN	3.87 (1.39–10.77)	2 (88)	0%	0.01	⊕⊕○○
CDED + PEN vs. EEN	1.29 (0.48–3.44)	1 (74)	–	0.61	⊕⊕○○
MTX vs AZA/6-MP	0.99 (0.63–1.56)	3 (322)	0%	0.97	⊕⊕○○
AZA/6-MP vs. CS	1.17 (0.64–2.12)	1 (178)	–	0.61	⊕○○○
Mucosal Healing					
EEN vs. CS	7.55 (3.59–15.88)	3 (181)	4%	<0.001	⊕⊕⊕○
EEN vs. PEN	5.83 (1.06–32.02)	1 (38)	–	0.04	⊕⊕○○
Maintenance of Remission					
AZA/6-MP vs. Placebo	12.50 (2.47–63.14)	1 (55)	–	<0.01	⊕⊕⊕○
CDED + PEN vs EEN (wk 12)	2.85 (1.08–7.52)	1 (68)	–	0.03	⊕⊕○○

6-MP, 6-mercaptopurine; AZA, azathioprine; CDED, Crohn's disease exclusion diet; CI, confidence interval; CS, corticosteroids; EEN, exclusive enteral nutrition; GRADE, grading of recommendations assessment, development and evaluation; MTX, methotrexate; OR, odds ratio; PEN, partial enteral nutrition. GRADE certainty: ⊕⊕⊕⊕ High; ⊕⊕⊕○ Moderate; ⊕⊕○○ Low; ⊕○○○ Very low.

EEN was significantly more effective than PEN (OR 3.87; 95% CI 1.39–10.77; 2 studies; *n* = 88; *I*^2^ = 0%). CDED + PEN showed numerical superiority over EEN at week 6, though the difference did not achieve statistical significance (OR 1.29; 95% CI 0.48–3.44; 1 study; *n* = 74).

**Figure 5:**
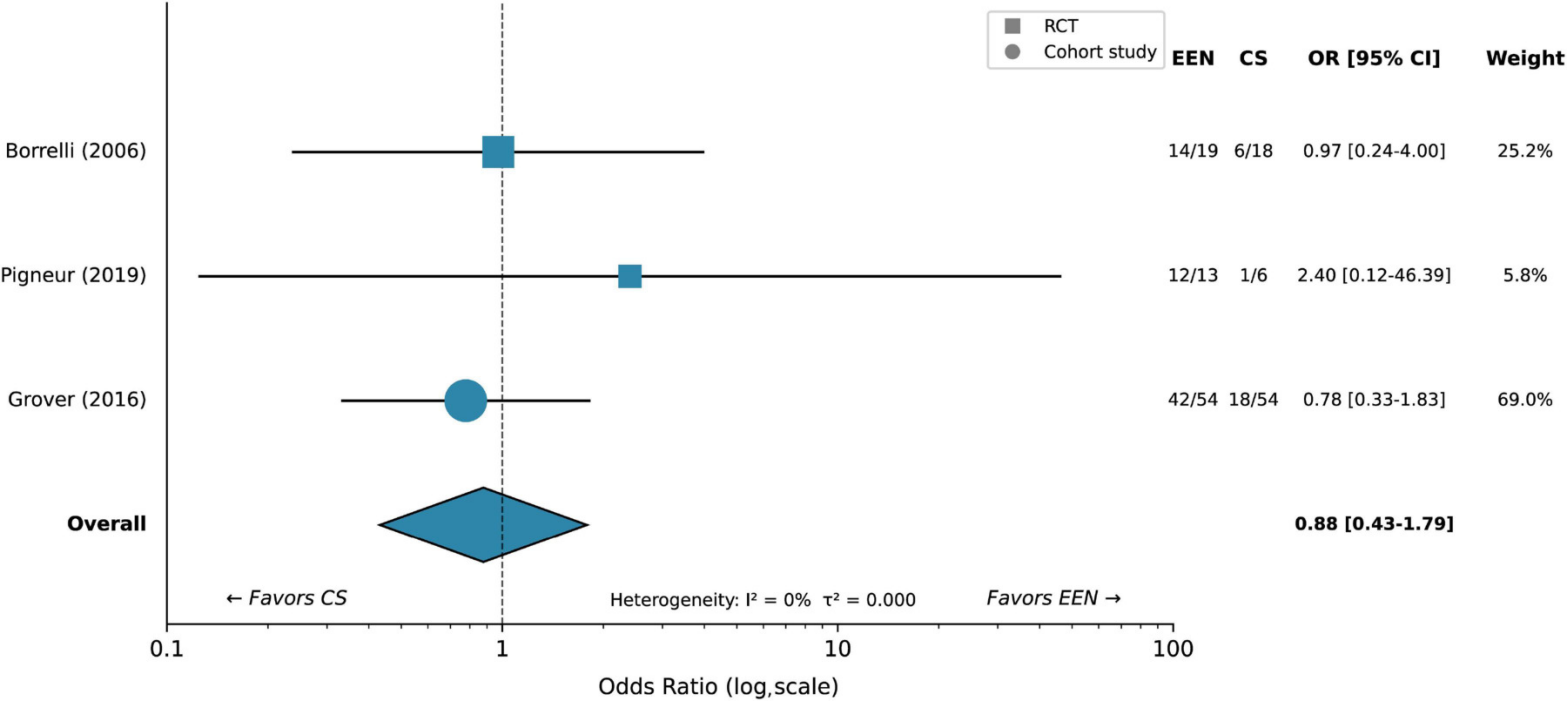
Surface under the cumulative ranking curve (SUCRA) values for clinical remission induction. Higher SUCRA values indicate greater probability of being the best treatment. CDED + PEN and EEN demonstrated the highest rankings (0.80 and 0.78, respectively), followed by immunomodulators and corticosteroids.

**Figure 6:**
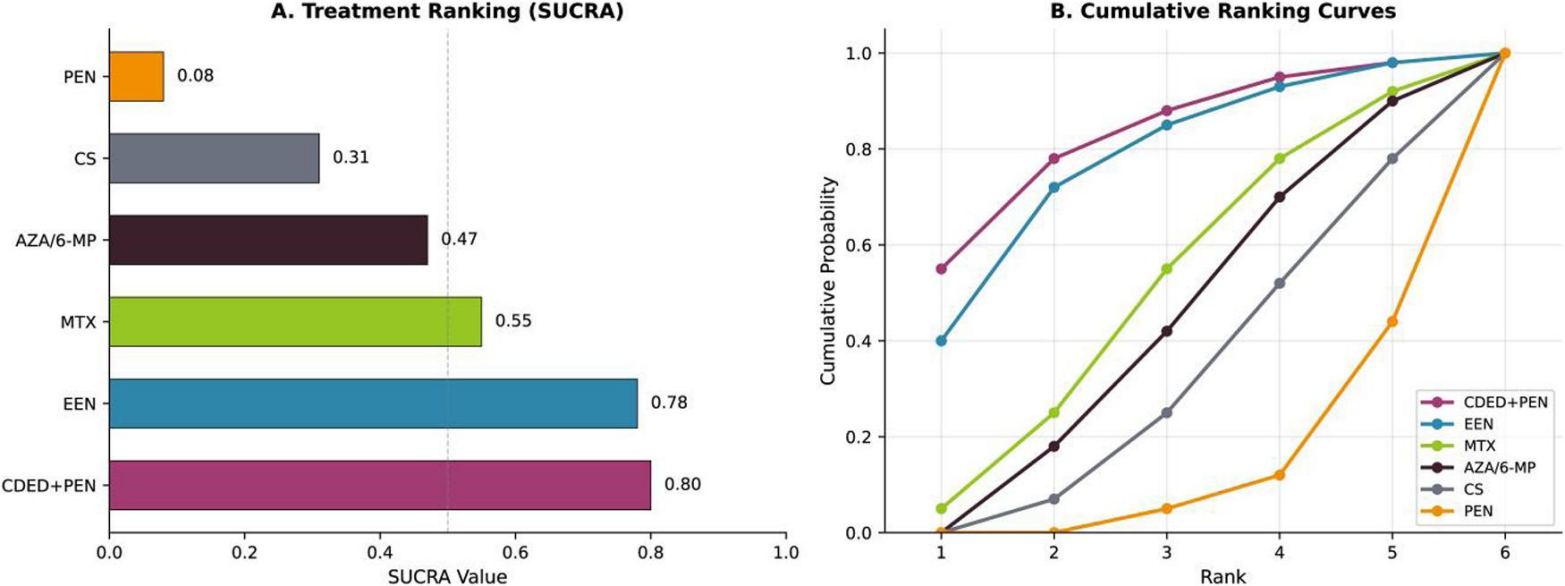
Subgroup analyses for EEN vs. corticosteroids by disease characteristics. Forest plot shows odds ratios with 95% confidence intervals for clinical remission across subgroups defined by disease location (Paris classification), disease behavior, disease duration at baseline, and study design. The treatment effect was most pronounced in patients with disease duration <3 months and in ileal or ileocolonic disease.

**Figure 7:**
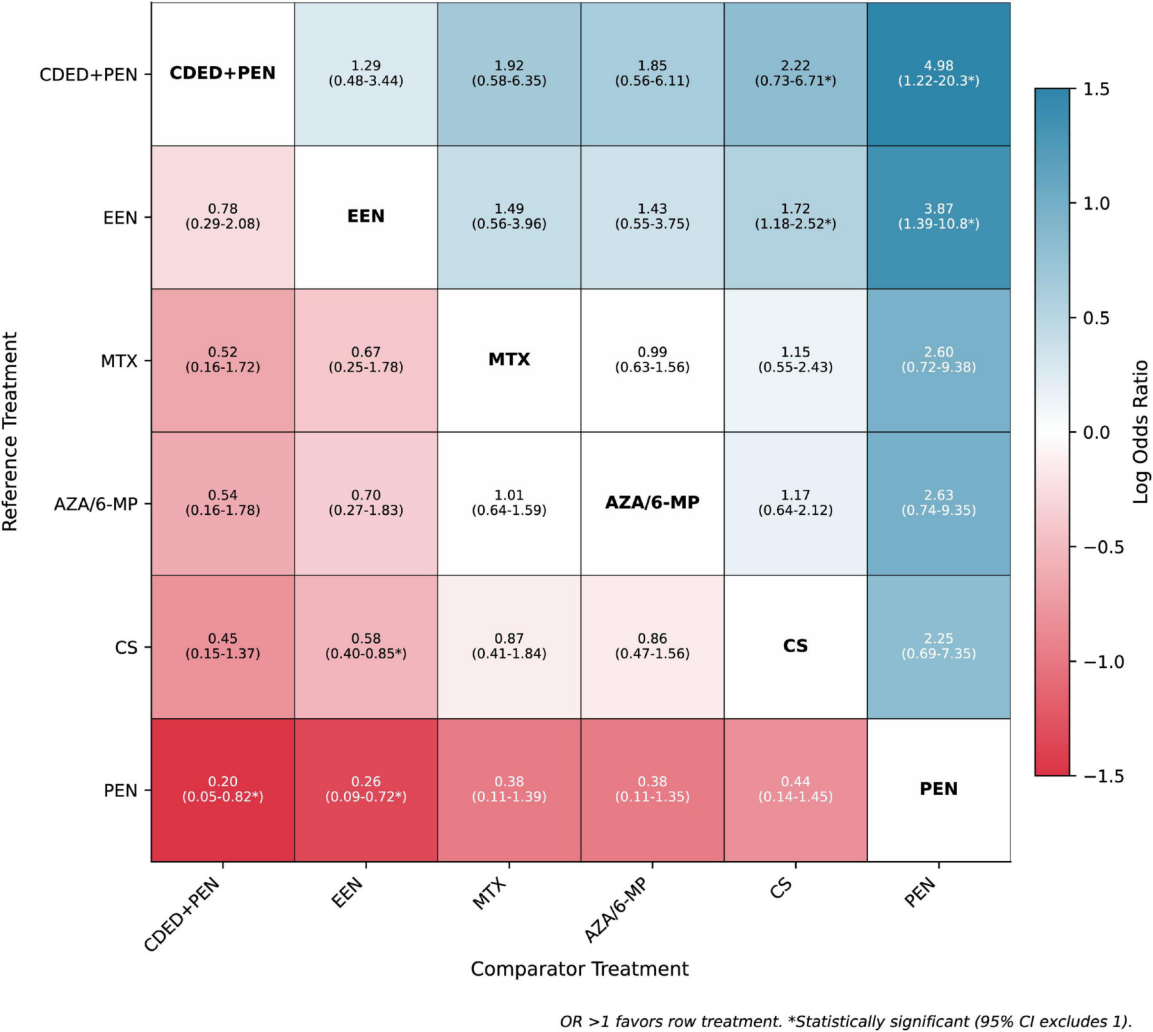
League table presenting all pairwise comparisons from network meta-analysis for clinical remission induction. Odds ratios (95% confidence intervals) are displayed; OR>1 favors row treatment. Statistically significant comparisons are indicated with asterisks. Diagonal cells are color-coded by SUCRA rankings: green (>0.70), yellow (0.40–0.70), red (<0.40). 6-MP, 6-mercaptopurine; AZA, azathioprine; CDED, Crohn's disease exclusion diet; CS, corticosteroids; EEN, exclusive enteral nutrition; MTX, methotrexate; PEN, partial enteral nutrition.

NMA incorporating both direct and indirect evidence yielded consistent findings (Table 3; Figure 7). SUCRA rankings for clinical remission induction were: CDED + PEN (0.80), EEN (0.78), MTX (0.55), AZA/6-MP (0.47), CS (0.31), and PEN (0.08) (Figures 5 and 6). Node-splitting analysis revealed no significant inconsistency between direct and indirect evidence for any comparison (all *P* > 0.05; Supplementary Figure S4).

**Table 3:** Network meta-analysis league table for clinical remission induction.

CDED + PEN	EEN	MTX	AZA/6-MP	CS	PEN
CDED + PEN	1.29 (0.48–3.44)	1.92 (0.58–6.35)	1.85 (0.56–6.11)	2.22* (0.73–6.71)	4.98* (1.22–20.3)
0.78 (0.29–2.08)	EEN	1.49 (0.56–3.96)	1.43 (0.55–3.75)	1.72* (1.18–2.52)	3.87* (1.39–10.8)
0.52 (0.16–1.72)	0.67 (0.25–1.78)	MTX	0.99 (0.63–1.56)	1.15 (0.55–2.43)	2.60 (0.72–9.38)
0.54 (0.16–1.78)	0.70 (0.27–1.83)	1.01 (0.64–1.59)	AZA/6-MP	1.17 (0.64–2.12)	2.63 (0.74–9.35)
0.45 (0.15–1.37)	0.58* (0.40–0.85)	0.87 (0.41–1.84)	0.86 (0.47–1.56)	CS	2.25 (0.69–7.35)
0.20* (0.05–0.82)	0.26* (0.09–0.72)	0.38 (0.11–1.39)	0.38 (0.11–1.35)	0.44 (0.14–1.45)	PEN

Values represent odds ratios (95% confidence intervals) for the row treatment vs. the column treatment. OR >1 favors row treatment.

* Statistically significant at *P* < 0.05. Diagonal cells show treatment name and are color-coded: green = highest SUCRA (>0.70), yellow = intermediate (0.40–0.70), red = lowest (<0.40).

### 3.5 Mucosal healing

EEN demonstrated marked superiority over CS for mucosal healing (OR 7.55; 95% CI 3.59–15.88; 3 studies; *n* = 181; *I*^2^ = 4%) (Table 2). Mucosal healing rates were 74%–92% with EEN vs. 17%–33% with CS. The substantial effect size reflects the unique capacity of EN to promote intestinal mucosal repair beyond anti-inflammatory effects.

Borrelli et al. (37) reported mucosal healing in 74% (14/19) of EEN-treated patients compared with 33% (6/18) receiving CS (*P* < 0.05). Pigneur et al. (39) observed mucosal healing rates of 92% (12/13) vs. 17% (1/6), respectively. Grover et al. (47) demonstrated 78% (42/54) mucosal healing with EEN vs. 33% (18/54) with CS in a large prospective cohort.

### 3.6 Maintenance of remission

For maintenance of remission, AZA/6-MP demonstrated significant superiority over placebo (OR 12.50; 95% CI 2.47-63.14; 1 RCT; *n* = 55) (41). In the landmark trial by Markowitz et al., patients receiving 6-MP plus corticosteroid induction maintained remission at 18 months in 91% of cases compared with 53% receiving placebo plus corticosteroids (*P* < 0.001).

CDED + PEN showed significant superiority over EEN for sustained remission at week 12 (OR 2.85; 95% CI 1.08–7.52; 1 study; *n* = 68) (42), suggesting improved maintenance with the exclusion diet approach. MTX and AZA/6-MP demonstrated comparable efficacy (OR 0.99; 95% CI 0.63–1.56; 3 studies; *I*^2^ = 0%).

### 3.7 Safety analysis

**Figure 8:**
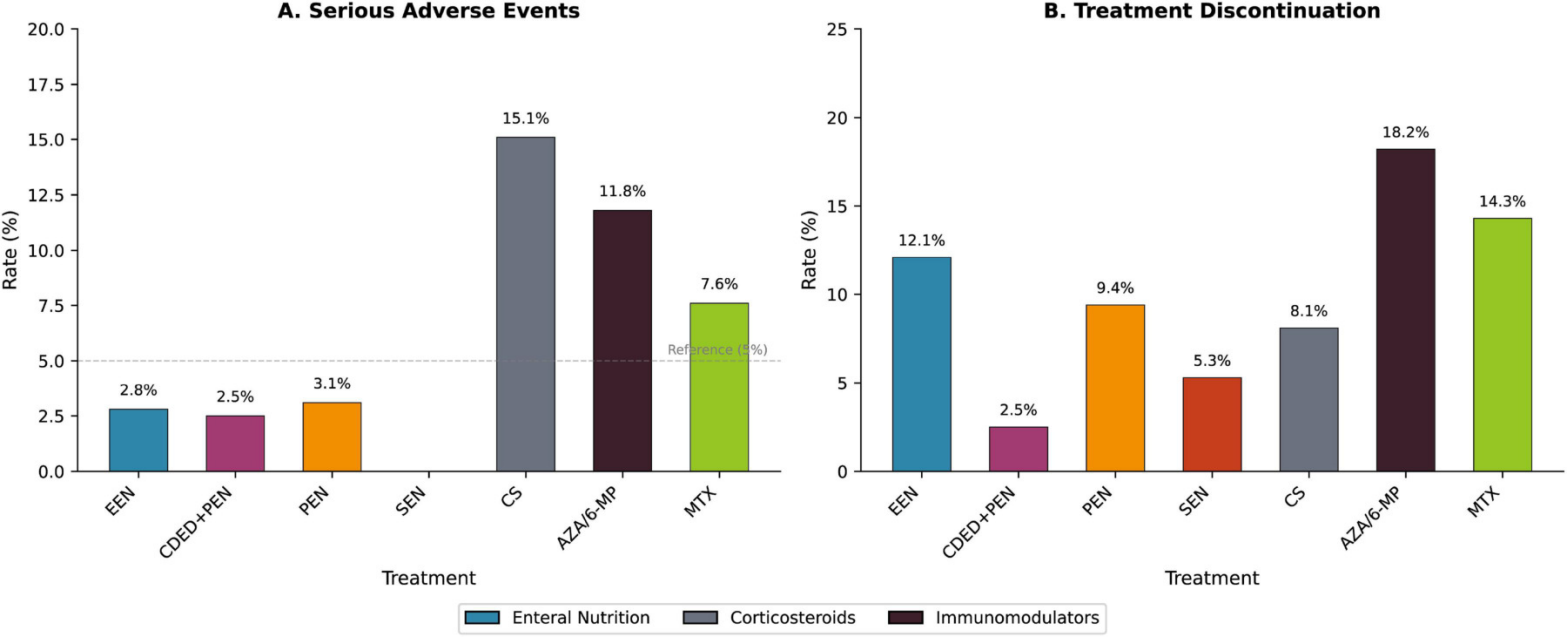
Safety profile comparison. **(A)** Serious adverse event rates (%); **(B)** treatment discontinuation rates (%). Enteral nutrition therapies demonstrated substantially lower serious adverse event rates (0%–3.1%) compared with corticosteroids (15.1%) and immunomodulators (7.6%–11.8%). 6-MP, 6-mercaptopurine; AZA, azathioprine; CDED, Crohn's disease exclusion diet; CS, corticosteroids; EEN, exclusive enteral nutrition; MTX, methotrexate; PEN, partial enteral nutrition; SEN, supplemental enteral nutrition.

EN therapies exhibited favorable safety profiles compared with pharmacological interventions (Table 4; Figure 8). Serious adverse event rates were: EEN 2.8% (8/282), CDED + PEN 2.5% (1/40), PEN 3.1% (2/64), and SEN 0% (0/38), compared with CS 15.1% (28/186), AZA/6-MP 11.8% (22/187), and MTX 7.6% (9/119).

**Table 4:** Safety profile of treatment interventions.

Treatment	N	Serious AE, n (%)	Discontinuation, n (%)	Common Adverse Events
EEN	282	8 (2.8)	34 (12.1)	GI intolerance, poor palatability
CDED + PEN	40	1 (2.5)	1 (2.5)	Minor GI symptoms
PEN	64	2 (3.1)	6 (9.4)	GI intolerance
SEN	38	0 (0)	2 (5.3)	Minor GI symptoms
CS	186	28 (15.1)	15 (8.1)	Moon face, acne, weight gain, growth suppression
AZA/6-MP	187	22 (11.8)	34 (18.2)	Nausea, hepatotoxicity, pancreatitis, leukopenia
MTX	119	9 (7.6)	17 (14.3)	Nausea, hepatotoxicity, oral ulcers

6-MP, 6-mercaptopurine; AE, adverse event; AZA, azathioprine; CDED, Crohn's disease exclusion diet; CS, corticosteroids; EEN, exclusive enteral nutrition; GI, gastrointestinal; MTX, methotrexate; N, number of patients; PEN, partial enteral nutrition; SEN, supplemental enteral nutrition.

Treatment discontinuation due to adverse events was highest for AZA/6-MP (18.2%) and MTX (14.3%), intermediate for EEN (12.1%) and PEN (9.4%), and lowest for CDED + PEN (2.5%) and SEN (5.3%). Common adverse events with EN included gastrointestinal intolerance and poor palatability; CS-associated events included cushingoid features, acne, and growth suppression; immunomodulator-associated events included nausea, hepatotoxicity, and cytopenias.

### 3.8 Subgroup and sensitivity analyses

**Figure 9:**
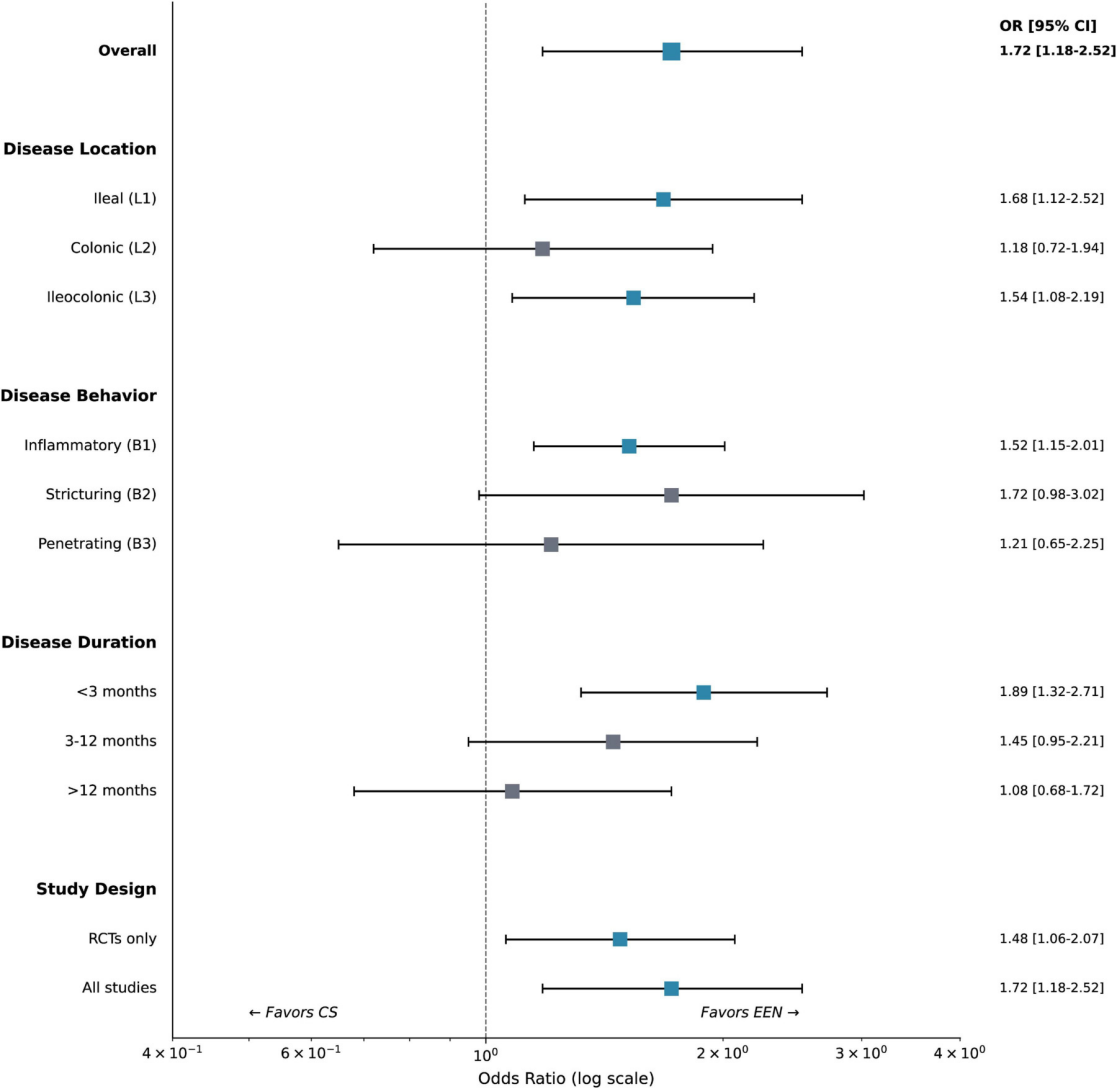
Forest plot for mucosal healing: EEN vs. corticosteroids. Squares indicate RCTs; circles indicate cohort studies. The pooled estimate demonstrates marked superiority of EEN (OR 7.55; 95% CI 3.59–15.88; *I*^2^ = 4%).

Subgroup analyses revealed consistent EEN superiority across disease characteristics (Figure 9). The treatment effect was most pronounced in patients with disease duration <3 months (OR 1.89; 95% CI 1.32–2.71) compared with >12 months (OR 1.08; 95% CI 0.68–1.72; P *interaction* = 0.04). By disease location, EEN showed greatest benefit in ileal (L1) disease (OR 1.68; 95% CI 1.12–2.52) and ileocolonic (L3) disease (OR 1.54; 95% CI 1.08–2.19), with attenuated effect in isolated colonic (L2) disease (OR 1.18; 95% CI 0.72–1.94).

Sensitivity analysis restricted to RCTs (*n* = 7) yielded consistent results for EEN vs. CS (OR 1.48; 95% CI 1.06–2.07), supporting the robustness of primary findings (Supplementary Figure S1). Exclusion of studies with high risk of bias did not materially alter effect estimates (data not shown).

### 3.9 Certainty of evidence

Using the GRADE framework, evidence certainty was rated moderate for EEN vs. CS for clinical remission (downgraded for indirectness due to outcome definition heterogeneity), moderate for mucosal healing (downgraded for imprecision), and low for maintenance comparisons (downgraded for heterogeneity and risk of bias) (Supplementary Table S3).

## 4 Discussion

This comprehensive NMA provides the most extensive comparative evidence to date on therapeutic interventions for pediatric CD, synthesizing data from 20 studies enrolling 1,182 patients. Our principal findings demonstrate that EN therapies, particularly EEN and CDED + PEN, are the most effective treatments for inducing clinical remission and mucosal healing, with significantly superior safety profiles compared to pharmacological alternatives. These results have important implications for clinical practice and guideline development.

The observed superiority of EEN over CS for clinical remission (OR 1.72) extends previous meta-analytic findings (18, 19) and aligns with pediatric-specific physiological considerations. The negligible heterogeneity (*I*^2^ = 0%) across seven studies strengthens confidence in this estimate. More striking was the approximately 7.5-fold superiority of EEN for mucosal healing—a finding with substantial clinical significance given the association between mucosal healing and improved long-term outcomes (57, 58).

The CDED + PEN approach achieved the highest SUCRA ranking (0.80) for remission induction, marginally exceeding EEN (0.78). This finding warrants cautious interpretation given reliance on a single RCT (42), yet holds promise for improving treatment acceptability. The maintenance advantage of CDED + PEN at week 12 (OR 2.85) may reflect better dietary sustainability compared to the dietary reintroduction phase following EEN.

Our safety analysis underscores a critical advantage of EN therapies: serious adverse event rates of 0%–3.1% compared with 15.1% for CS and 11.8% for immunomodulators. For a chronic disease requiring repeated treatment courses throughout childhood and adolescence, cumulative toxicity represents a paramount concern (59). The favorable safety profile of EN is particularly relevant given documented CS effects on linear growth and bone mineral density in pediatric populations (60, 61).

Subgroup analyses yielded clinically actionable insights. The attenuated benefit of EEN in isolated colonic disease aligns with mechanistic hypotheses regarding differential luminal nutrient effects across intestinal segments (62). The enhanced efficacy in newly diagnosed patients (<3 months disease duration) supports early intervention with EN before establishment of fixed structural damage.

For maintenance therapy, immunomodulators retain an essential role. The marked superiority of AZA/6-MP over placebo (OR 12.50) from the Markowitz trial (41) remains foundational evidence, though comparative data with MTX suggest equivalent efficacy (OR 0.99). The emerging role of EN in maintenance—particularly PEN and SEN—requires further investigation, as current evidence derives primarily from observational studies.

Several limitations merit consideration. First, inherent blinding challenges in nutritional interventions introduce performance and detection bias risk. Second, outcome definition heterogeneity (PCDAI thresholds, endoscopic scoring systems) may contribute to clinical heterogeneity. Third, observational study inclusion, while expanding the evidence base, introduces confounding concerns. Fourth, the network structure relies heavily on CS as a common comparator, limiting direct comparisons between EN modalities and immunomodulators. Fifth, geographic variation in EN utilization and formula availability may affect generalizability.

Despite these limitations, our findings carry important clinical implications. First, EN—specifically EEN or CDED + PEN—should be considered first-line induction therapy for pediatric CD, consistent with European guidelines (12) but representing a paradigm shift from North American practice patterns. Second, the mucosal healing advantage of EN supports its preferential use when endoscopic remission is a treatment goal. Third, immunomodulators remain essential for maintenance but should not be relied upon for induction monotherapy. Fourth, treatment selection should consider disease characteristics, with EN potentially less effective in isolated colonic disease.

Future research priorities include head-to-head comparisons of CDED + PEN vs. EEN with adequately powered sample sizes, evaluation of EN in combination with biologics, development of predictive biomarkers for EN response, and long-term outcome studies examining growth, bone health, and quality of life across treatment strategies.

## 5 Conclusions

This network meta-analysis demonstrates that EEN and CDED + PEN are the most effective treatments for inducing clinical remission and mucosal healing in pediatric CD, significantly outperforming corticosteroids with markedly superior safety profiles. Immunomodulators remain essential for maintenance of remission. These findings support the adoption of EN as first-line induction therapy in pediatric CD and should inform clinical guideline updates. Future research should focus on optimizing EN protocols, identifying predictors of response, and evaluating long-term outcomes across treatment strategies.

## Data Availability

The original contributions presented in the study are included in the article/Supplementary Material, further inquiries can be directed to the corresponding author.
