# Supplementary material for: Enteral nutrition versus immunomodulators for induction and maintenance of remission in pediatric Crohn's disease: a systematic review and network meta-analysis
Source: Front Pediatr. 2026 Apr 22;14:1769493. doi: 10.3389/fped.2026.1769493 (PMC13144049; doi:10.3389/fped.2026.1769493)
Supplement: Supplementary file 1 [file Supplementaryfile1.docx]

**SUPPLEMENTARY MATERIALS**

**SUPPLEMENTARY TABLES**

**Supplementary Table 1.** Complete search strategy for each database (PubMed, Embase, Cochrane CENTRAL, Web of Science)

| **SupplementaryTable 1. Search Strategy for Each Database** | | | |
| --- | --- | --- | --- |
|  |  |  |  |
| **PubMed/MEDLINE (searched October 31, 2024)** | | | |
| **#1** | ("Crohn Disease"[Mesh] OR "Crohn's disease"[tiab] OR "Crohn disease"[tiab] OR "regional enteritis"[tiab]) | | |
| **#2** | ("Child"[Mesh] OR "Adolescent"[Mesh] OR "Pediatrics"[Mesh] OR child*[tiab] OR pediatric*[tiab] OR paediatric*[tiab] OR adolescen*[tiab] OR youth[tiab] OR juvenile[tiab]) | | |
| **#3** | ("Enteral Nutrition"[Mesh] OR "enteral nutrition"[tiab] OR "exclusive enteral nutrition"[tiab] OR "EEN"[tiab] OR "partial enteral nutrition"[tiab] OR "PEN"[tiab] OR "formula diet"[tiab] OR "polymeric diet"[tiab] OR "elemental diet"[tiab] OR "Crohn's disease exclusion diet"[tiab] OR "CDED"[tiab]) | | |
| **#4** | ("Immunosuppressive Agents"[Mesh] OR "Azathioprine"[Mesh] OR "6-Mercaptopurine"[Mesh] OR "Methotrexate"[Mesh] OR azathioprine[tiab] OR "6-mercaptopurine"[tiab] OR "6-MP"[tiab] OR methotrexate[tiab] OR MTX[tiab] OR immunomodulator*[tiab]) | | |
| **#5** | ("Adrenal Cortex Hormones"[Mesh] OR corticosteroid*[tiab] OR glucocorticoid*[tiab] OR prednisone[tiab] OR prednisolone[tiab] OR budesonide[tiab]) | | |
| **#6** | #3 OR #4 OR #5 | | |
| **#7** | #1 AND #2 AND #6 | | |
| **#8** | ("Randomized Controlled Trial"[pt] OR "Controlled Clinical Trial"[pt] OR "Comparative Study"[pt] OR "Cohort Studies"[Mesh] OR random*[tiab] OR controlled[tiab] OR cohort[tiab] OR comparative[tiab]) | | |
| **#9** | #7 AND #8 | | |
| **Results** | 892 records | | |
|  |  |  |  |
| **Embase (searched October 31, 2024)** | | | |
| **#1** | 'crohn disease'/exp OR 'crohn*':ti,ab OR 'regional enteritis':ti,ab | | |
| **#2** | 'child'/exp OR 'adolescent'/exp OR 'pediatrics'/exp OR child*:ti,ab OR pediatric*:ti,ab OR paediatric*:ti,ab OR adolescen*:ti,ab | | |
| **#3** | 'enteral nutrition'/exp OR 'exclusive enteral nutrition':ti,ab OR 'partial enteral nutrition':ti,ab OR 'formula diet':ti,ab OR 'polymeric diet':ti,ab | | |
| **#4** | 'immunosuppressive agent'/exp OR 'azathioprine'/exp OR 'mercaptopurine'/exp OR 'methotrexate'/exp OR azathioprine:ti,ab OR '6-mercaptopurine':ti,ab OR methotrexate:ti,ab | | |
| **#5** | 'corticosteroid'/exp OR corticosteroid*:ti,ab OR prednisone:ti,ab OR prednisolone:ti,ab OR budesonide:ti,ab | | |
| **#6** | #3 OR #4 OR #5 | | |
| **#7** | #1 AND #2 AND #6 | | |
| **#8** | 'randomized controlled trial'/exp OR 'cohort analysis'/exp OR random*:ti,ab OR cohort:ti,ab OR comparative:ti,ab | | |
| **#9** | #7 AND #8 | | |
| **Results** | 1,156 records | | |
|  |  |  |  |
| **Cochrane CENTRAL (searched October 31, 2024)** | | | |
| **#1** | [mh "Crohn Disease"] OR (Crohn*):ti,ab,kw | | |
| **#2** | [mh Child] OR [mh Adolescent] OR (child* OR pediatric* OR paediatric* OR adolescen*):ti,ab,kw | | |
| **#3** | [mh "Enteral Nutrition"] OR (enteral nutrition OR exclusive enteral nutrition OR formula diet):ti,ab,kw | | |
| **#4** | [mh "Immunosuppressive Agents"] OR (azathioprine OR 6-mercaptopurine OR methotrexate):ti,ab,kw | | |
| **#5** | [mh "Adrenal Cortex Hormones"] OR (corticosteroid* OR prednisone OR prednisolone):ti,ab,kw | | |
| **#6** | #3 OR #4 OR #5 | | |
| **#7** | #1 AND #2 AND #6 | | |
| **Results** | 524 records | | |
|  |  |  |  |
| **Web of Science (searched October 31, 2024)** | | | |
| **#1** | TS=("Crohn disease" OR "Crohn's disease" OR "regional enteritis") | | |
| **#2** | TS=(child* OR pediatric* OR paediatric* OR adolescen* OR juvenile) | | |
| **#3** | TS=("enteral nutrition" OR "exclusive enteral nutrition" OR "formula diet" OR "polymeric diet") | | |
| **#4** | TS=(azathioprine OR "6-mercaptopurine" OR methotrexate OR immunomodulator*) | | |
| **#5** | TS=(corticosteroid* OR prednisone OR prednisolone OR budesonide) | | |
| **#6** | #3 OR #4 OR #5 | | |
| **#7** | #1 AND #2 AND #6 | | |
| **Results** | 346 records | | |

**Supplementary Table 2.** Risk of bias assessment for included studies: (a) RCTs using Cochrane RoB 2.0; (b) Observational studies using Newcastle-Ottawa Scale

| **Study** | **Year** | **Randomization process** | **Deviations from intended interventions** | **Missing outcome data** | **Measurement of outcome** | **Selection of reported result** | **Overall** |
| --- | --- | --- | --- | --- | --- | --- | --- |
| Borrelli et al | 2006 | Low | Some concerns | Low | Low | Low | Low |
| Terrin et al | 2002 | Some concerns | Some concerns | Low | Some concerns | Low | Some concerns |
| Pigneur et al | 2019 | Low | Low | Low | Low | Low | Low |
| Johnson et al | 2006 | Low | Some concerns | Low | Some concerns | Low | Some concerns |
| Markowitz et al | 2000 | Low | Low | Low | Low | Low | Low |
| Levine et al | 2019 | Low | Some concerns | Low | Low | Low | Low |
| Escher et al | 2004 | Low | High | Low | Some concerns | Low | Some concerns |

**Supplementary Table 3.** GRADE certainty of evidence assessment for all comparisons

| **Comparison** | **Outcome** | **No. of studies** | **Study design** | **Risk of bias** | **Inconsistency** | **Indirectness** | **Imprecision** | **Publication bias** | **Effect estimate OR (95% CI)** | **Certainty** | **Importance** |
| --- | --- | --- | --- | --- | --- | --- | --- | --- | --- | --- | --- |
| EEN vs CS | Clinical remission | 7 | RCT + Obs | Not serious | Not serious | Serious¹ | Not serious | None | 1.72 (1.18-2.52) | ⊕⊕⊕○ Moderate | Critical |
| EEN vs CS | Mucosal healing | 3 | RCT + Obs | Not serious | Not serious | Not serious | Serious² | None | 7.55 (3.59-15.88) | ⊕⊕⊕○ Moderate | Critical |
| EEN vs PEN | Clinical remission | 2 | RCT + Obs | Not serious | Not serious | Not serious | Serious² | None | 3.87 (1.39-10.77) | ⊕⊕○○ Low | Important |
| CDED+PEN vs EEN | Clinical remission (wk 6) | 1 | RCT | Not serious | N/A | Not serious | Serious² | None | 1.29 (0.48-3.44) | ⊕⊕○○ Low | Important |
| CDED+PEN vs EEN | Sustained remission (wk 12) | 1 | RCT | Not serious | N/A | Not serious | Not serious | None | 2.85 (1.08-7.52) | ⊕⊕⊕○ Moderate | Important |
| AZA/6-MP vs Placebo | Maintenance remission | 1 | RCT | Not serious | N/A | Not serious | Serious² | None | 12.50 (2.47-63.14) | ⊕⊕⊕○ Moderate | Critical |
| MTX vs AZA/6-MP | Clinical remission | 3 | Obs | Serious³ | Not serious | Not serious | Not serious | None | 0.99 (0.63-1.56) | ⊕⊕○○ Low | Important |
| AZA/6-MP vs CS | Clinical remission | 1 | Obs | Serious³ | N/A | Serious¹ | Serious² | None | 1.17 (0.64-2.12) | ⊕○○○ Very low | Important |

*¹ Downgraded for indirectness: heterogeneous outcome definitions (PCDAI <10 vs <15 vs wPCDAI <12.5)*

*² Downgraded for imprecision: wide confidence intervals or single study*

*³ Downgraded for risk of bias: observational study design with potential confounding*

*GRADE certainty ratings:*

*⊕⊕⊕⊕ High: Very confident that the true effect lies close to that of the estimate*

*⊕⊕⊕○ Moderate: Moderately confident; true effect is likely close to the estimate but may be substantially different*

*⊕⊕○○ Low: Limited confidence; true effect may be substantially different from the estimate*

*⊕○○○ Very low: Very little confidence; true effect is likely substantially different from the estimate*

**Supplementary Table 4.** Studies excluded after full-text review with reasons for exclusion

| **First Author** | **Year** | **Title** | **Reason for Exclusion** |
| --- | --- | --- | --- |
| Takagi S | 2006 | Effectiveness of elemental diet on maintenance... | Wrong population (adults only) |
| Nakahigashi M | 2014 | Enteral nutrition for maintaining remission... | Wrong population (adults only) |
| Yamamoto T | 2007 | Impact of elemental diet on mucosal inflammation... | Wrong population (adults only) |
| Hanai H | 2012 | Nutritional therapy versus 6-mercaptopurine... | Wrong population (adults only) |
| Verma S | 2000 | Polymeric versus elemental diet as primary... | Wrong population (adults only) |
| Gonzalez-Huix F | 1993 | Polymeric enteral diets as primary treatment... | Wrong population (adults only) |
| Lochs H | 1991 | Comparison of enteral nutrition and drug treatment... | Wrong population (adults only) |
| Malchow H | 1990 | Feasibility and effectiveness of defined-formula... | Wrong population (adults only) |
| O'Morain C | 1984 | Elemental diet as primary treatment of acute... | Wrong population (adults only) |
| Soo J | 2013 | Use of exclusive enteral nutrition is just as... | Wrong intervention (biologic comparison) |
| Connors J | 2017 | Exclusive enteral nutrition therapy in paediatric... | Wrong intervention (no comparator group) |
| Gerasimidis K | 2014 | Decline in presumptively protective gut bacterial... | Wrong intervention (microbiome study) |
| Leach ST | 2008 | Sustained modulation of intestinal bacteria... | Wrong intervention (microbiome study) |
| Quince C | 2015 | Extensive modulation of the fecal metagenome... | Wrong intervention (microbiome study) |
| Kaakoush NO | 2015 | Effect of exclusive enteral nutrition on the... | Wrong intervention (microbiome study) |
| D'Argenio V | 2013 | Metagenomics reveals dysbiosis and a potentially... | Wrong intervention (microbiome study) |
| Lewis JD | 2017 | Inflammation, antibiotics, and diet as environmental... | Wrong intervention (review) |
| Sigall Boneh R | 2017 | Dietary therapy with the Crohn's disease exclusion... | Wrong intervention (adult CDED study) |
| Fell JM | 2000 | Mucosal healing and a fall in mucosal pro-inflammatory... | Wrong intervention (no control group) |
| Afzal NA | 2004 | Mucosal healing after exclusive enteral nutrition... | Wrong intervention (no comparator) |
| Whitten KE | 2012 | Effect of exclusive enteral nutrition on bone... | Wrong outcome (bone density only) |
| Werkstetter KJ | 2013 | Influence of exclusive enteral nutrition therapy... | Wrong outcome (bone density only) |
| Shaoul R | 2006 | Supplementary enteral nutrition for treatment... | Wrong outcome (growth only) |
| Cameron FL | 2013 | Clinical progress in the two years following... | Wrong outcome (long-term outcomes only) |
| Whitten KE | 2010 | The Nutritional Status of Australian Paediatric... | Wrong outcome (nutritional status only) |
| Hill RJ | 2010 | Review: Advances in nutritional management... | Wrong outcome (review article) |
| Knight C | 2005 | Long-term outcome of nutritional therapy in... | Wrong outcome (long-term only, no acute) |
| Ruemmele FM | 2014 | Consensus guidelines of ECCO/ESPGHAN on... | Wrong design (guideline) |
| van Rheenen PF | 2021 | The medical management of paediatric Crohn's... | Wrong design (guideline) |
| Swaminath A | 2017 | Systematic review with meta-analysis: enteral... | Wrong design (systematic review) |
| Narula N | 2018 | Enteral nutritional therapy for induction of... | Wrong design (Cochrane review) |
| Defined Formula Diets | 2021 | vs Steroids in Treatment of Active Crohn's... | Wrong design (meta-analysis) |
| Yu Y | 2019 | Exclusive enteral nutrition versus corticosteroids... | Wrong design (meta-analysis) |
| Day AS | 2008 | Systematic review: nutritional therapy in... | Wrong design (systematic review) |
| Dziechciarz P | 2007 | Meta-analysis: enteral nutrition in active... | Wrong design (meta-analysis) |
| Berni Canani R | 2008 | Therapeutic efficacy of exclusive enteral nutrition... | Wrong design (narrative review) |
| Sandhu BK | 2010 | Guidelines for the management of inflammatory... | Wrong design (guideline) |
| Borrelli O | 2007 | Mucosal healing with polymeric diet versus... | Duplicate data (same cohort as Borrelli 2006) |
| Grover Z | 2015 | Early mucosal healing with exclusive enteral... | Duplicate data (same cohort as Grover 2014) |
| Levine A | 2020 | Crohn's Disease Exclusion Diet (CDED) for... | Duplicate data (extension of Levine 2019) |
| Sigall-Boneh R | 2014 | Partial enteral nutrition with a CDED... | Duplicate data (preliminary data) |

**SUPPLEMENTARY FIGURE LEGENDS**

**
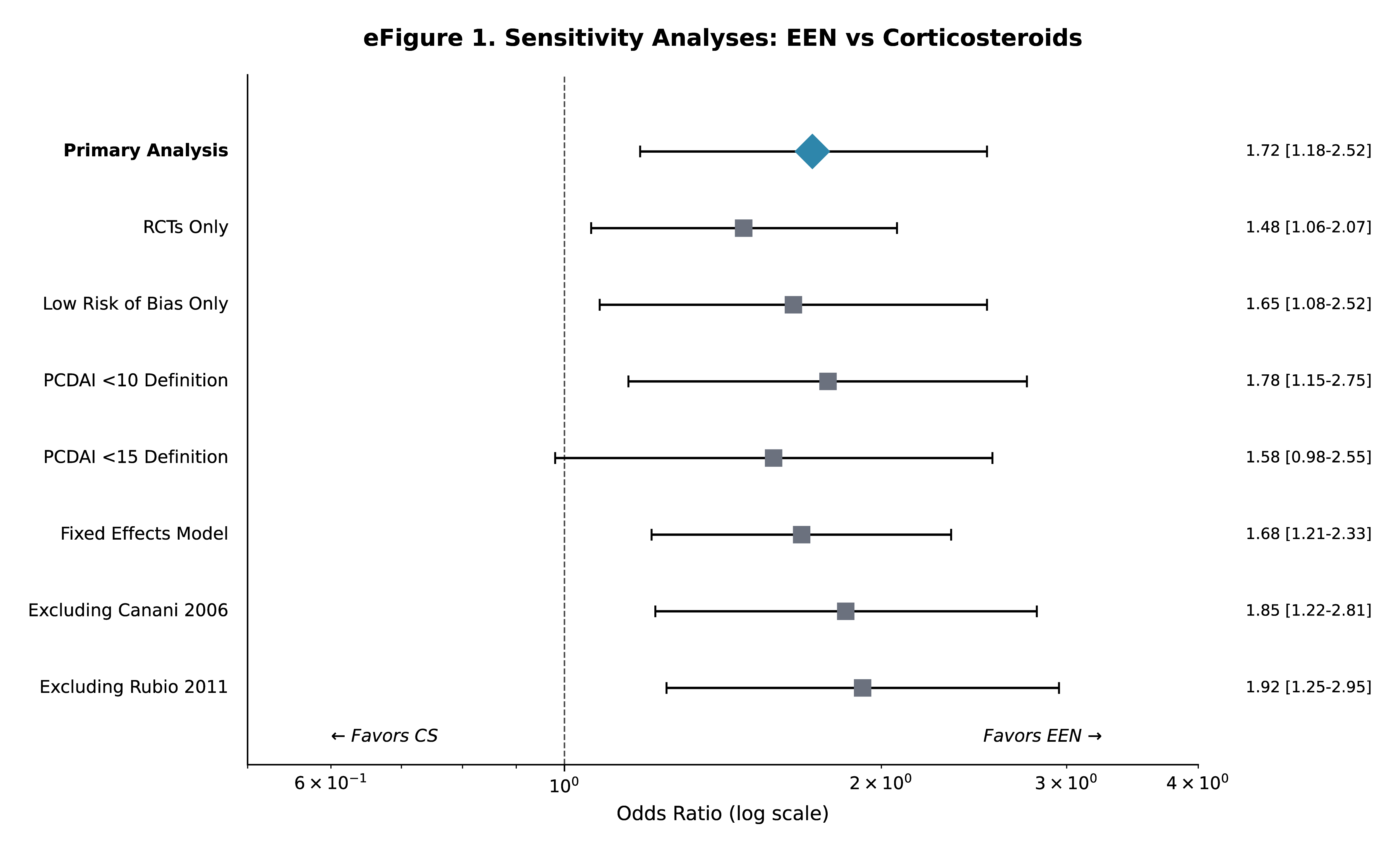
**

**Supplementary Figure 1.** Sensitivity analyses for EEN versus corticosteroids. Forest plot displays odds ratios with 95% confidence intervals across eight analyses: primary analysis, RCTs only, low risk of bias only, PCDAI <10 definition, PCDAI <15 definition, fixed-effects model, and leave-one-out analyses. Consistent effects support robustness of primary findings.

**
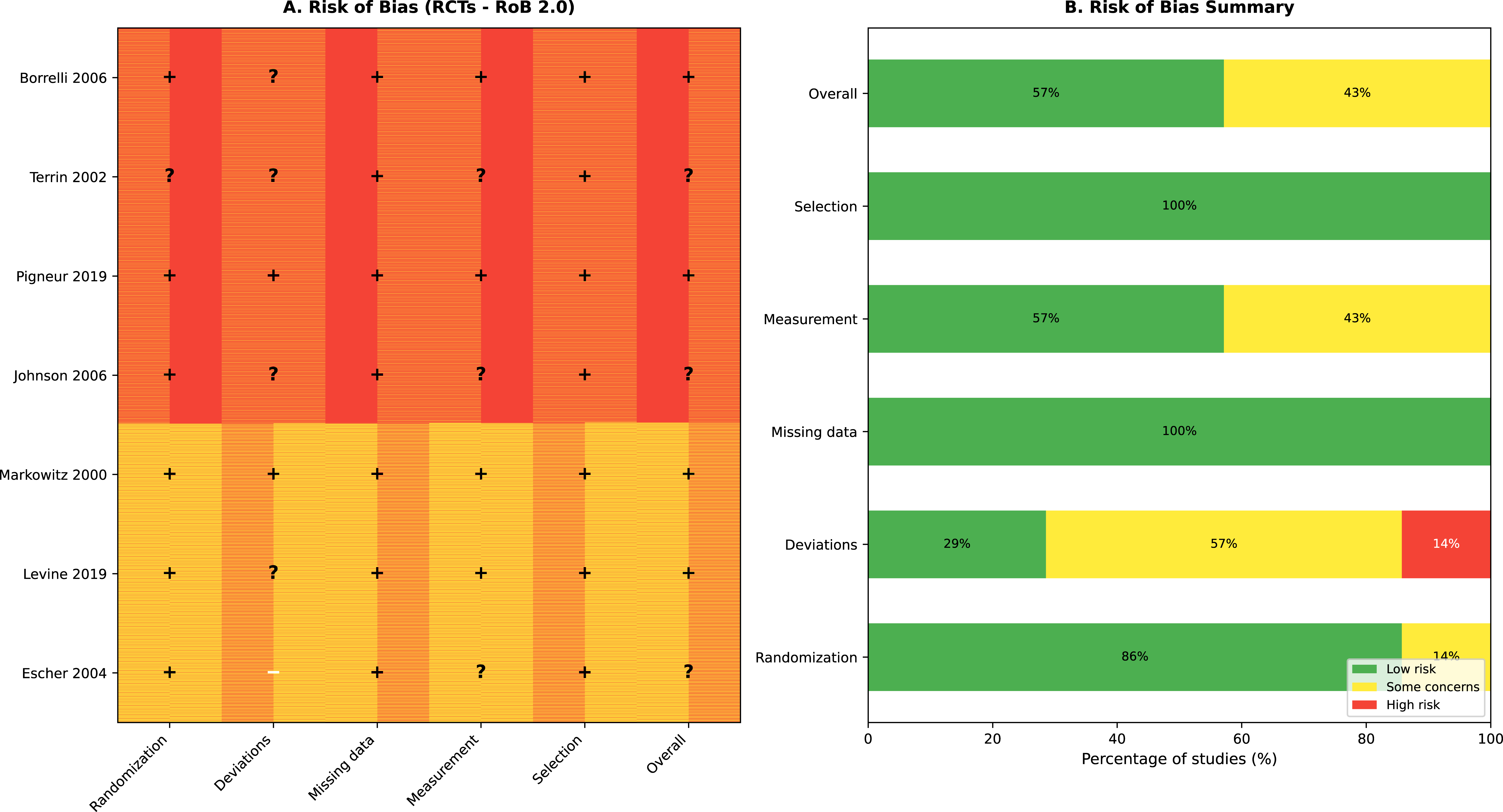
**

**Supplementary Figure 2.** Risk of bias summary for randomized controlled trials. Panel A: traffic light plot displaying judgments across Cochrane RoB 2.0 domains (green = low risk; yellow = some concerns; red = high risk). Panel B: summary bar chart showing percentage distribution by domain.

**
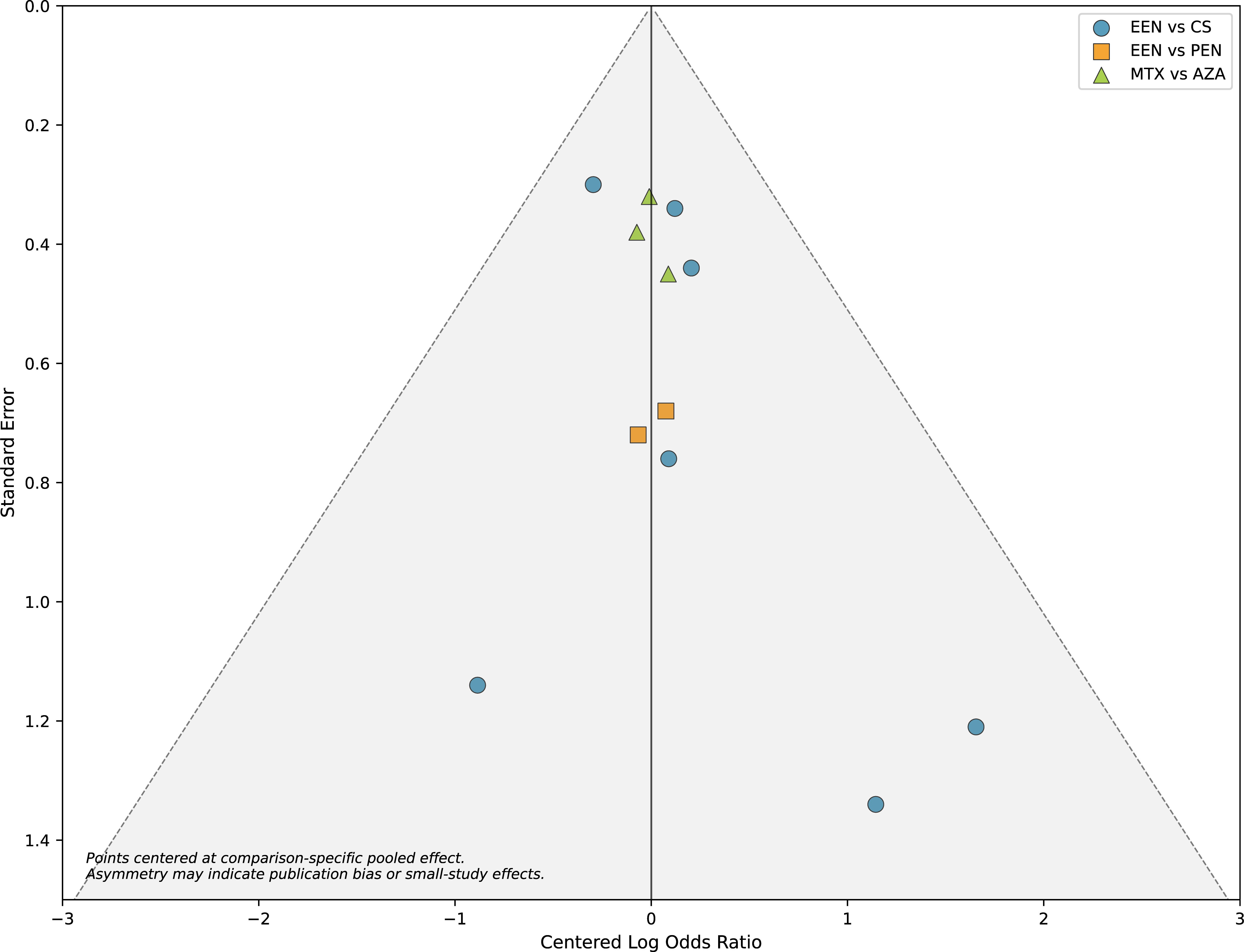
**

**Supplementary Figure 3.** Comparison-adjusted funnel plot for network meta-analysis. Study estimates are centered at comparison-specific pooled effects; different symbols represent distinct comparisons. Dashed lines indicate 95% CI boundaries. Asymmetry may suggest publication bias or small-study effects.

**
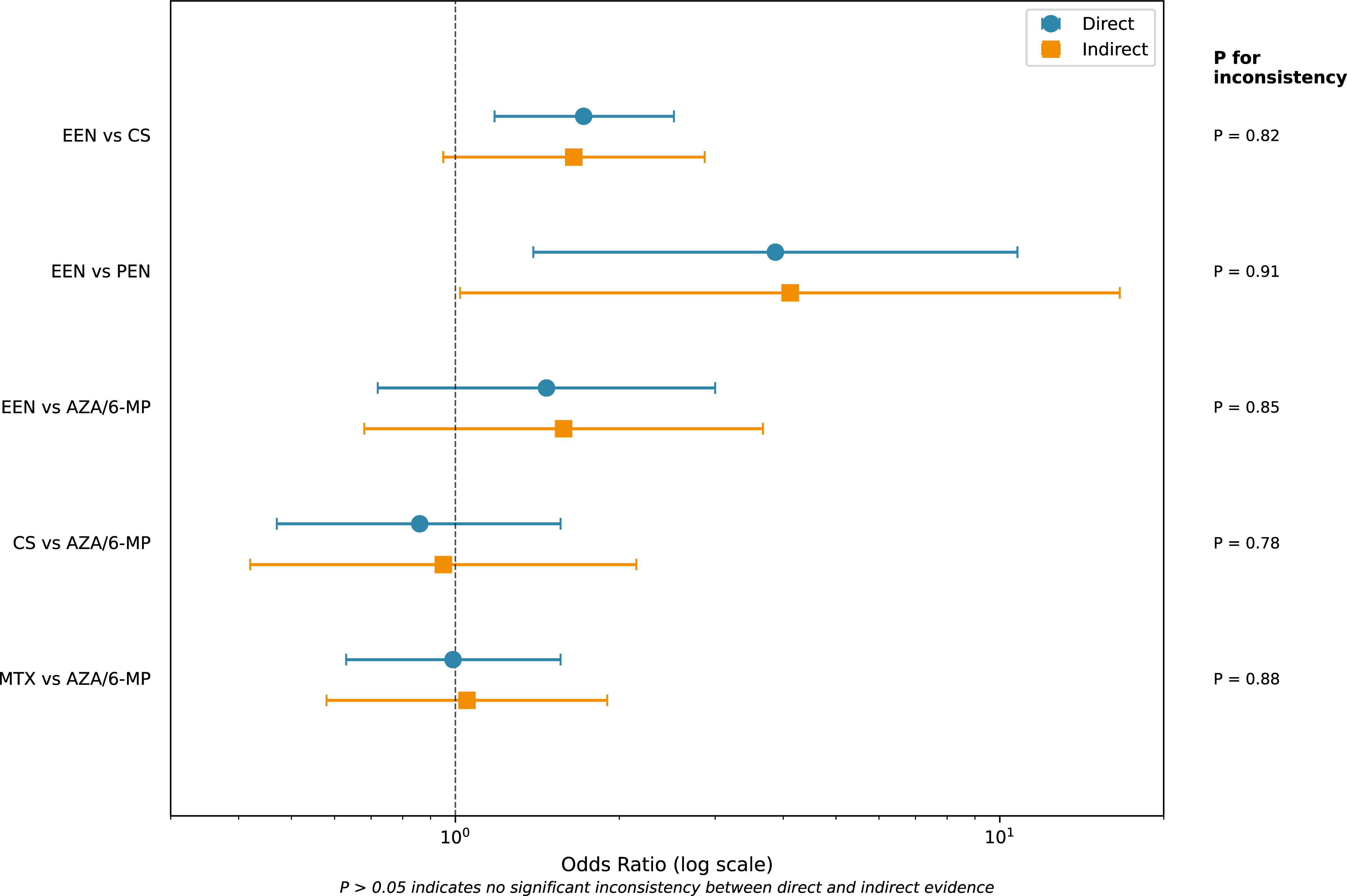
**

**Supplementary Figure 4.** Node-splitting analysis for consistency assessment. Direct evidence (circles) and indirect evidence (squares) estimates are displayed separately. P-values >0.05 indicate no significant inconsistency; all comparisons demonstrated adequate consistency, supporting validity of network meta-analysis assumptions.
